# Neurofunctional Correlates of Ethical, Food-Related Decision-Making

**DOI:** 10.1371/journal.pone.0120541

**Published:** 2015-04-01

**Authors:** J. Bradley C. Cherry, Jared M. Bruce, Jayson L. Lusk, John M. Crespi, Seung-Lark Lim, Amanda S. Bruce

**Affiliations:** 1 Department of Psychology, the University of Missouri–Kansas City, Kansas City, Missouri, United States of America; 2 Department of Agricultural Economics, Oklahoma State University, Stillwater, Oklahoma, United States of America; 3 Department of Agricultural Economics, Kansas State University, Manhattan, Kansas, United States of America; 4 Department of Psychology, the University of Missouri–Kansas City, Kansas City, Missouri, United States of America; 5 Joint Department of Pediatrics, the University of Kansas Medical Center and Children’s Mercy Hospital, Kansas City, Kansas, United States of America

## Abstract

For consumers today, the perceived ethicality of a food’s production method can be as important a purchasing consideration as its price. Still, few studies have examined how, neurofunctionally, consumers are making ethical, food-related decisions. We examined how consumers’ ethical concern about a food’s production method may relate to how, neurofunctionally, they make decisions whether to purchase that food. Forty-six participants completed a measure of the extent to which they took ethical concern into consideration when making food-related decisions. They then underwent a series of functional magnetic resonance imaging (fMRI) scans while performing a food-related decision-making (FRDM) task. During this task, they made 56 decisions whether to purchase a food based on either its price (i.e., high or low, the “price condition”) or production method (i.e., with or without the use of cages, the “production method condition”), but not both. For 23 randomly selected participants, we performed an exploratory, whole-brain correlation between ethical concern and differential neurofunctional activity in the price and production method conditions. Ethical concern correlated negatively and significantly with differential neurofunctional activity in the left dorsolateral prefrontal cortex (dlPFC). For the remaining 23 participants, we performed a confirmatory, region-of-interest (ROI) correlation between the same variables, using an 8-mm3 volume situated in the left dlPFC. Again, the variables correlated negatively and significantly. This suggests, when making ethical, food-related decisions, the more consumers take ethical concern into consideration, the less they may rely on neurofunctional activity in the left dlPFC, possibly because making these decisions is more routine for them, and therefore a more perfunctory process requiring fewer cognitive resources.

## Introduction

The foods we eat are changing, and with them, how we make decisions about those foods [1–2]. Over the last six decades, revolutionary forms of intensive animal agriculture have steadily supplanted the farms of yesterday with the feedlots of today [3]. Innovations to traditional methods of food production, together with the expansion, specialization, and vertical integration of the agricultural industry, have brought tremendous economic benefits to producers and consumers alike [3–5]. Today, more food is available [3, 6–7], and its production more economical [8–12], than ever before. But these benefits have not come without costs [13].

The growth of intensive animal agriculture—particularly the proliferation of concentrated animal feeding operations (CAFOs)—has also wrought serious consequences in the form of external costs, both to the environment and to the welfare of the animals concerned [4, 14–17]. A typical CAFO may emit into the environment a variety of gaseous, liquid, and particulate pollutants [4, 18–19]. In addition to the deleterious effects these pollutants have on local air and water quality [18–22], they contribute measurably to global warming [23–25] and endanger public health [26–29]. CAFOs also jeopardize the welfare of the animals raised therein [30–31]. Animals raised in CAFOs are forced to develop at faster rates, in smaller spaces, and in greater numbers than they would under less intensive circumstances [32]. These conditions conspire to increase the incidence of death and disease among the animals, in part by decreasing, even eliminating their access to natural environments, as well as their ability to perform natural behaviors [32–35].

External costs such as these have been characterized as presenting the most controversial [36–37], most publicized [38–39] issue facing animal agriculture today, a sentiment consumers appear to share [40]. In a study examining consumers’ willingness to pay for pork labeled with various “public good attributes,” Lusk, Nilsson, and Foster [41] found participants were willing to pay premiums of $0.67 per pound for pork labeled with an “environmental certification,” $0.84 per pound for pork labeled with an “animal welfare certification,” and $0.90 per pound for pork labeled with an “antibiotic certification.” In a more recent study, Norwood and Lusk [42] found participants were willing to pay premiums as high as $0.95 per dozen for eggs produced using an “aviary, pasture system,” as opposed to a “cage system,” and $1.01 per pound for pork produced using a “pasture system,” as opposed to a “crate system.” These results and those of similar studies (e.g., [39, 43–45]) provide compelling evidence of consumers’ ethical concern about the use of cages, crates, and other forms of confinement common in CAFOs. So substantial is this concern that they are willing to pay premiums for foods produced using less controversial methods. But while these results may offer an explanation for why consumers are making these decisions, they do less to explain *how*.

Examining this question through the lens of decision neuroscience may offer new insights into how consumers are making ethical, food-related decisions (see [46]). Although few studies have examined this question narrowly, many have examined the neurofunctional correlates of decision-making generally (e.g., [47–51]), and others, ethical (e.g., [52–55]) or food-related (e.g., [56–57]) decision-making specifically. Decision-making has been described as a circuital process requiring sensory input, prospective valuation, behavioral output, and retrospective valuation [49]. Of these constituent components, those related to valuation have formed the focus of many neurofunctional studies of decision-making [58]. The results of these studies have revealed the representation of value in decision-making contexts to rely generally on neurofunctional activity in the posterior parietal and prefrontal cortices [48, 59] (see also [60–61]), but specifically on that in the dorsolateral prefrontal cortex (dlPFC) and ventromedial prefrontal cortex (vmPFC) [62–64].

The same cortical regions are also thought to facilitate valuation in the more nuanced context of ethical decision-making. In a study examining, in part, the neurofunctional correlates of “personal and impersonal [ethical] judgment,” Greene, Nystrom, Engell, Darley, and Cohen [65] found impersonal ethical judgment correlated with differential neurofunctional activity in the dlPFC bilaterally, but particularly in the right dlPFC. In another study examining the effects of damage to the vmPFC on “utilitarian [ethical] judgement,” Koenigs et al. [66] found participants with such damage made decisions demonstrating a reliably, but unusually strong preference for the welfare of a group, as opposed to that of an individual, even when the decision made would require participants to sacrifice that individual’s life actively and directly.

Similar results have been found suggesting the dlPFC and vmPFC also facilitate valuation in the context of food-related decision-making. In a study examining the neurofunctional correlates of valuation during food-related decision-making, Plassmann, O’Doherty, and Rangel [67] found participants’ valuation of both appetitive and aversive foods correlated with differential neurofunctional activity in the right dlPFC. In another study examining self-control during food-related decision-making, Hare, Camerer, and Rangel [68] found participants’ valuation of foods in a decision-making context correlated with differential neurofunctional activity in the left dlPFC, as well as the vmPFC, regardless of participants’ levels of self-control (see also [69]).

The purpose of the present study was to expand on these findings, examining the neurofunctional correlates of ethical, food-related decision-making by exploring how consumers’ ethical concern about a food’s production method may relate to how, neurofunctionally, they make decisions whether to purchase that food. To this end, we examined the relationship between (a) the extent to which participants took ethical concern into consideration when making food-related decisions and (b) their differential neurofunctional activity when making decisions whether to purchase a food based on either its price or production method. Specifically, we examined how participants’ scores on the Ethical Concern subscale of the Food Choice Questionnaire (FCQ) [70] related to their differential neurofunctional activity when making binary, incentive-compatible, non-hypothetical decisions whether to purchase eggs differing in either their price (i.e., high or low) or production method (i.e., with or without the use of cages), but not both. We hypothesized participants’ scores on the Ethical Concern subscale of the FCQ would correlate positively and significantly with their differential neurofunctional activity, specifically in the dlPFC and vmPFC, when making decisions whether to purchase eggs based on their production method, as opposed to their price.

## Materials and Methods

The present study was approved by the Social Sciences Institutional Review Board of the University of Missouri–Kansas City (UMKC), as well as the Human Subjects Committee of the University of Kansas Medical Center (KUMC). All participants provided their written, informed consent to participate, the procedure for which was also approved by the aforementioned institutions.

### Participants

Forty-six healthy adults (*n*_female_ = 24), ranging in age from 21 to 55 years (*M* = 29.65 years, *SD* = 9.49 years), participated in the study. However, not all participants’ behavioral and neurofunctional data were included in subsequent analyses. Of the 46 participants who participated, one failed to maintain his head positioning while his neurofunctional data were being acquired, rendering half those data unsuitable for subsequent analyses. In addition to the 46 participants who participated, four others participated, but because they failed to follow instructions provided for performing the task administered as their neurofunctional data were being acquired, both their behavioral and neurofunctional data were excluded from subsequent analyses.

All participants were recruited from the Kansas City, Missouri, metropolitan area; were English speakers; had normal or corrected-to-normal vision; and were right-handed. None self-reported current use of psychotropic medication, current or past substance abuse, diagnosis of severe neuro- or psychopathology (e.g., epilepsy, schizophrenia, etc.), lactose intolerance, or vegan or vegetarian diet. Participants’ body masses ranged from 18.4 to 50.1 kg/m^2^ (*M* = 26.6 kg/m^2^, *SD* = 6.0 kg/m^2^). Education levels were self-reported as “high school” (*n* = 1 [2%]), “associate’s degree or some college” (*n* = 7 [15%]), “bachelor’s degree” (*n* = 27 [59%]), and “graduate degree” (*n* = 11 [24%]). Annual household incomes, also self-reported, were less than $20,000 per year (*n* = 18 [39%]), between $20,000 and $39,999 per year (*n* = 13 [28%]), between $40,000 and $59,999 per year (*n* = 6 [13%]), between $60,000 and $79,999 per year (*n* = 3 [7%]), between $80,000 and $99,999 per year (*n* = 2 [4%]), between $100,000 and $119,999 per year (*n* = 1 [2%]), and greater than $120,000 per year (*n* = 3 [7%]).

### Behavioral Data Measurement

To measure the extent to which participants took ethical concern into consideration when making food-related decisions, the FCQ [70] was administered. The FCQ is a 36-item, multidimensional measure of respondents’ self-reported tendency to take various factors into consideration when making decisions whether to consume a food. Each item on the FCQ describes a food-related attribute (e.g., “is packaged in an environmentally friendly way”) associated with one of the FCQ’s nine subscales: Convenience, Ethical Concern, Familiarity, Health, Mood, Natural Content, Price, Sensory Appeal, and Weight Control. Respondents rate each attribute using a four-point Likert scale ranging from one, “not at all important,” to four, “very important.” The values of these responses are then added to produce a score on each of the FCQ’s subscales. The higher a respondent’s score on a subscale, the more importance that respondent places on the factor corresponding with that subscale. Therefore, the higher a respondent’s score on the Ethical Concern subscale, the more that respondent takes ethical concern into consideration when making food-related decisions.

### Neurofunctional Data Acquisition

After completing the FCQ, each participant underwent a series of magnetic resonance imaging (MRI) scans, including one anatomical scan and two functional (fMRI) scans. These scans were performed at KUMC’s Hoglund Brain Imaging Center on a 3-T Magnetom Skyra scanner (Siemens, Erlangen, Germany). Following the acquisition of automated scout images to determine the orientation of the participant’s head relative to the scanner, as well as the performance of shimming procedures to optimize the homogeneity of the scanner’s electromagnetic field, the anatomical scan was performed. For this scan, T1-weighted, three-dimensional, magnetization-prepared, rapid acquisition with gradient-echo (MPRAGE) anatomical images were acquired (repetition time [TR] = 2,300 ms, echo time [TE] = 2 ms, flip angle = 9°, field of view [FoV] = 256 x 256 mm, matrix = 256 x 256 mm, in-plane resolution = 1 x 1 mm, gap thickness = 0 mm, slice thickness = 1 mm). Following this scan, the two functional scans were performed. For these scans, T2-weighted, gradient-echo, blood oxygenation level-dependent (BOLD) functional images were acquired in 50 contiguous, oblique, axial slices at a 40° angle (TR = 3,000 ms, TE = 25 ms, flip angle = 90°, FoV = 232 x 232 mm, matrix = 80 x 80 mm, in-plane resolution = 2.9 x 2.9 mm, gap thickness = 0 mm, slice thickness = 3 mm).

To optimize signal detection in the potential regions of interest (ROIs) in the present study (viz., the dlPFC and vmPFC, again, cortical regions responsible for, in part, the representation of value in decision-making contexts), and to minimize the presence of susceptibility artifacts, each participant’s head was carefully positioned to ensure the angle of the anterior commissure-posterior commissure (AC-PC) plane fell between 17° and 22° in the scanner’s coordinate space. This careful positioning ensured the 40° angle of acquisition was applied uniformly during all scans (see [71]). To maintain this positioning, and to facilitate signal reception and transmission, a 12-channel head coil was used to stabilize the participant’s head during the scans.

### Experimental Paradigm

While undergoing the two functional scans, participants performed a food-related decision-making (FRDM) task of event-related design (see [72]). For this task, participants were instructed to make binary decisions whether to purchase different types of eggs. Specifically, they were instructed to “make a series of choices between two food products…. Please choose carefully, as you will receive one of the food products you choose at the end of the experiment.” In addition to guiding participants in their performance of the task, these instructions improved the task’s incentive-compatibility by informing participants their decision-making would be non-hypothetical. That is, for every decision they would make to purchase a particular type of eggs, there was a possibility they would actually receive those eggs after performing the task.

**Fig 1:**
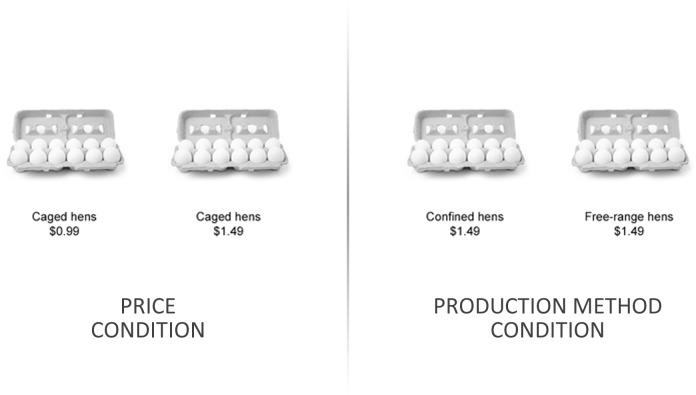
Examples of decisions from the price and production method conditions.

The task was presented on a screen positioned at the rear of the scanner’s bore. Participants viewed this screen using a mirror system attached to the head coil, and they used a control pad to make their decisions. For each decision, two options were presented, one on the left side of the screen, and one on the right. These options consisted of visually identical, one-dozen cartons of eggs, and below each appeared two attributes, one describing the option’s price (the “price attribute”), and one describing its production method (the “production method attribute”). The price attribute was either “$0.99,” “$1.49,” “$1.99,” “$2.49,” “$2.99,” “$3.49,” “$3.99,” or “$4.49,” (note the range of values for the price attribute was determined using historical data for the average price-per-dozen of eggs between 2004 and 2008 (see [37], p. 262) while the production method attribute was either “Caged hens,” “Cage-free hens,” “Confined hens,” or “Free-range hens.” For each decision, either the price attributes differed (the “price condition”), or the production method attributes differed (the “production method condition”), but not both (see Fig. 1). Also, each decision was presented twice, once with the options in one orientation, and later with the options in the opposite orientation.

**Fig 2:**
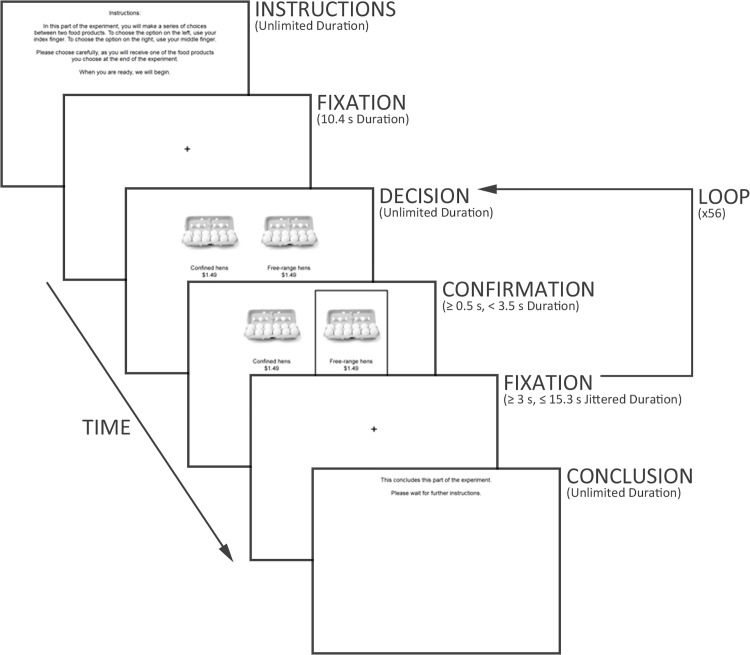
Schematic depicting the FRDM task.

In total, 56 decisions were presented, 28 from each of the two conditions. The order in which they were presented was randomized and counterbalanced across participants, and the intervals mediating their presentation were variably jittered in duration (see [72]). Each was presented until participants made a decision, at which time that decision was confirmed for 500 ms (see Fig. 2). However, if participants made a decision in less than 3 s, that decision was confirmed until 3 s had elapsed since the time the decision was presented, and then for an additional 500 ms. This encouraged participants to make their decisions at a deliberate, unhurried pace, and it ensured sufficient time was allowed for the acquisition of neurofunctional data as participants performed the task.

### Data Analyses

Behavioral and neurofunctional data were analyzed in BrainVoyager QX, version 2.4 (Brain Innovation, Maastricht, Netherlands), as well as Statistical Package for the Social Sciences (SPSS), version 21 (IBM, Armonk, United States). Before neurofunctional data were analyzed, they were first preprocessed using sinc-interpolated, trilinear, three-dimensional motion correction; three-dimensional spatial smoothing (full width at half maximum [FWHM] = 4 mm); and high-pass temporal filtering (sine-cosine cycles = 2). They were then coregistered through the transformation and translation of each functional image to align with its anatomical counterpart. These realigned images were then further transformed to conform to the spatial constraints defined by Talairach and Tournoux’s [73] co-planar, stereotaxic atlas, ensuring neurofunctional data were standardized in their spatial representation.

Neurofunctional data were analyzed using parametric statistical methods (see [74]). A multiple-regression analysis was performed using a random effects, general linear model, the results of which were used to generate, across all participants, a three-dimensional map depicting percent BOLD signal change between neurofunctional activity in the production method condition and that in the price condition (the “production method > price contrast”). Differential neurofunctional activity in this contrast was examined in order to isolate, to the extent possible, the effect on neurofunctional activity of requiring participants to make decisions whether to purchase a food based on its production method, as opposed to its price; that is, prompting them to take ethical concern into consideration as they made their decisions. Neurofunctional activity in the price and production method conditions was modeled as that occurring between the time at which each decision was first presented and the time at which a decision was made (participants’ “response times”). Regressors representing neurofunctional activity in the price and production method conditions were modeled using a hemodynamic response filter.

Following this multiple-regression analysis, for the purposes of subsequent analyses, participants were randomly assigned to two groups of 23 participants each. Between these groups, there were no statistically significant differences in age, *t*(44) = 0.46, *p* = 0.65, 95% CI [−4.39, 7.00]; sex, χ^2^(1, *N* = 46) = 0.35, *p* = 0.56; body mass, *t*(44) = 0.56, *p* = 0.58, 95% CI [−2.60, 4.60]; education level, *t*(44) = −0.42, *p* = 0.68, 95% CI [−0.51, 0.33]; annual household income, *t*(44) = −0.08, *p* = 0.93, 95% CI [−1.09, 1.01]; or score on the Ethical Concern subscale of the FCQ (see Results). There was also no statistically significant difference in response time to decisions in the price condition (*M* = 2.43 s, *SD* = 0.62 s), *t*(44) = 1.51, *p* = 0.14, 95% CI [−90.44, 636.10], nor to those in the production method condition (*M* = 2.38 s, *SD* = 0.61 s), *t*(44) = 1.01, *p* = 0.32, 95% CI [−180.17, 541.25]. Moreover, within the first group (“Group A”), there was no statistically significant difference in response time to decisions in the price condition (*M* = 2.57 s, *SD* = 0.47 s) and response time to decisions in the production method condition (*M* = 2.47 s, *SD* = 0.54 s), *t*(22) = 0.92, *p* = 0.37, 95% CI [−126.30, 328.02]. The same was true of the second group (“Group B”); the difference in response time to decisions in the price condition (*M* = 2.30 s, *SD* = 0.73 s) and response time to decisions in the production method condition (*M* = 2.29 s, *SD* = 0.67 s) was statistically insignificant, *t*(22) = 0.12, *p* = 0.91, 95% CI [−143.90, 161.03].

Using behavioral and neurofunctional data from participants in Group A, an exploratory, whole-brain correlation was performed for the purpose of identifying potential ROIs, particularly in the dlPFC and vmPFC. Because only one ROI was ultimately identified, specifically in the left dlPFC, that ROI then served as the location for a subsequent, confirmatory, ROI correlation using behavioral and neurofunctional data from participants in Group B. This conservative analytical approach allowed for an examination of the replicability of any correlations observed as a result of the whole-brain correlation, while also mitigating the implausibility or possible speciousness of any replicated correlations (see [75]).

For the whole-brain correlation, the results of the previously performed multiple-regression analysis were used to generate, for each participant in Group A, another three-dimensional map, this time consisting of beta weights representing differential neurofunctional activity in the production method > price contrast. Using these beta weights and participants’ scores on the Ethical Concern subscale of the FCQ, a Pearson correlation was then performed to identify clusters of statistically significant correlation. To correct for multiple comparisons (α < 0.05, *p* < 0.01), a Monte Carlo simulation was performed to determine a cluster-size threshold of 14 contiguous voxels. Clusters of correlation exceeding this threshold were considered statistically significant.

The subsequent, ROI correlation was performed in much the same manner as the whole-brain correlation, but was confined to an 8-mm^3^ volume situated in the left dlPFC, again, the only ROI identified as a result of the whole-brain correlation. Across this volume, for each participant in Group B, the average percent BOLD signal change in the production method > price contrast was extracted and imported into SPSS. A Pearson correlation was then performed using these average percent BOLD signal changes and participants’ scores on the Ethical Concern subscale of the FCQ.

## Results

### Behavioral Data

Participants’ scores on the Ethical Concern subscale of the FCQ ranged from 3 to 10 (*M* = 5.15, *SD* = 1.76). In Group A, they ranged from 4 to 10 (*M* = 5.61, *SD* = 1.85), and in Group B, from 3 to 8 (*M* = 4.70, *SD* = 1.58). Between these groups, there was no statistically significant difference in participants’ scores, *t*(44) = 1.80, *p* = 0.08, 95% CI [−0.11, 1.94].

### Neurofunctional Data

#### Whole-brain correlation

The whole-brain correlation was performed using only data from participants in Group A. In the production method > price contrast, participants’ scores on the Ethical Concern subscale of the FCQ correlated positively and significantly with their differential neurofunctional activity in eight cortical regions (see Table 1). That is, in the production method > price contrast, higher ethical concern when making food-related decisions correlated with higher differential neurofunctional activity in these cortical regions. Put more plainly, when making decisions whether to purchase eggs based on their production method, as opposed to their price, those participants who took ethical concern into higher consideration also demonstrated higher differential neurofunctional activity in these cortical regions.

**Table 1:** Results of the whole-brain and ROI correlations.

Cortical Regions	Coordinates^a^				*df*	Size^b^
	x	y	z	*r*		
**Whole-brain correlation (Group A)**						
(R) Precuneus, BA 7	17	−59	42	0.74**	22	17
(R) Lingual gyrus, BA 18	8	−65	6	0.82**	22	210
(R) Cingulate gyrus, BA 30	20	−56	18	0.77**	22	74
(L) Superior frontal gyrus, BA 9	−13	61	30	**−0.61** **	22	15
(L) Precuneus, BA 7	−13	−71	39	0.77**	22	101
(L) Middle occipital gyrus, BA 19	−31	−77	9	0.73**	22	211
(L) Postcentral gyrus, BA 3	−19	−32	69	0.68**	22	18
(L) Inferior parietal lobule, BA 40	−43	−29	48	0.70**	22	159
(L) Postcentral gyrus, BA 3	−49	−17	48	0.69**	22	17
**ROI correlation (Group B)**						
(L) Superior frontal gyrus, BA 9	−14	57	25^c^	**−0.46** *	22	8 mm^3^

Note. Correlations are between participants’ scores on the Ethical Concern subscale of the FCQ and their differential neurofunctional activity in the production method > price contrast. Correlation coefficients for the whole-brain correlation are provided for illustrative purposes only, and not, by themselves, as evidence of neurofunctional correlates (see [75]).

^a^Coordinates are provided, in Talairach convention [73], for the voxel of peak correlation in a cortical region, except for the ROI correlation, for which the coordinates of the center of the ROI are provided.

^b^Size is expressed in units of contiguous voxels unless otherwise specified.

^c^Although the coordinates of the center of the ROI differ slightly from those of the corresponding cortical region identified as a result of the whole-brain correlation (viz., the left superior frontal gyrus), this was to ensure no part of the ROI extended beyond the prefrontal cortex.

*p < 0.05.

**p < 0.01.

**Fig 3:**
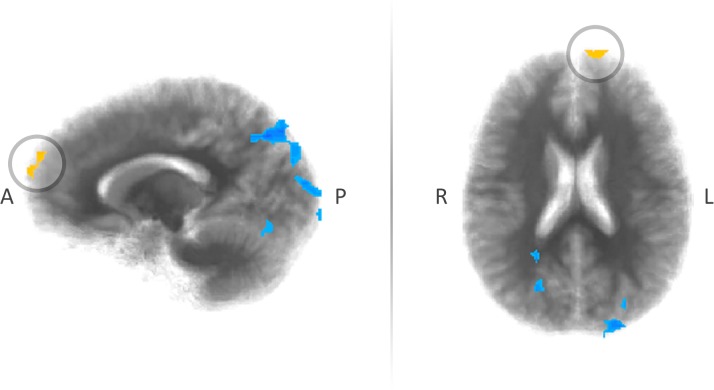
Sagittal and axial images depicting the cluster of negative correlation in the left dlPFC, specifically the left superior frontal gyrus, observed as a result of the whole-brain correlation. Images are depicted in radiological convention.

Conversely, in one other cortical region—specifically the left superior frontal gyrus, part of the left dlPFC—the same data were negatively and significantly correlated, *r*(21) = −0.61, *p* < 0.01 (see Table 1, Fig. 3; note the correlation coefficient for the whole-brain correlation is provided for illustrative purposes only, and not, by itself, as evidence of a neurofunctional correlate [see [75]]). Here, in the production method > price contrast, higher ethical concern when making food-related decisions actually correlated with *lower* differential neurofunctional activity. Again, more plainly, when making decisions whether to purchase eggs based on their production method, as opposed to their price, those participants who took ethical concern into higher consideration here demonstrated lower differential neurofunctional activity. Because this cortical region was the only ROI identified as a result of the whole-brain correlation, it then served as the location for the subsequent, ROI correlation.

#### ROI correlation

**Fig 4:**
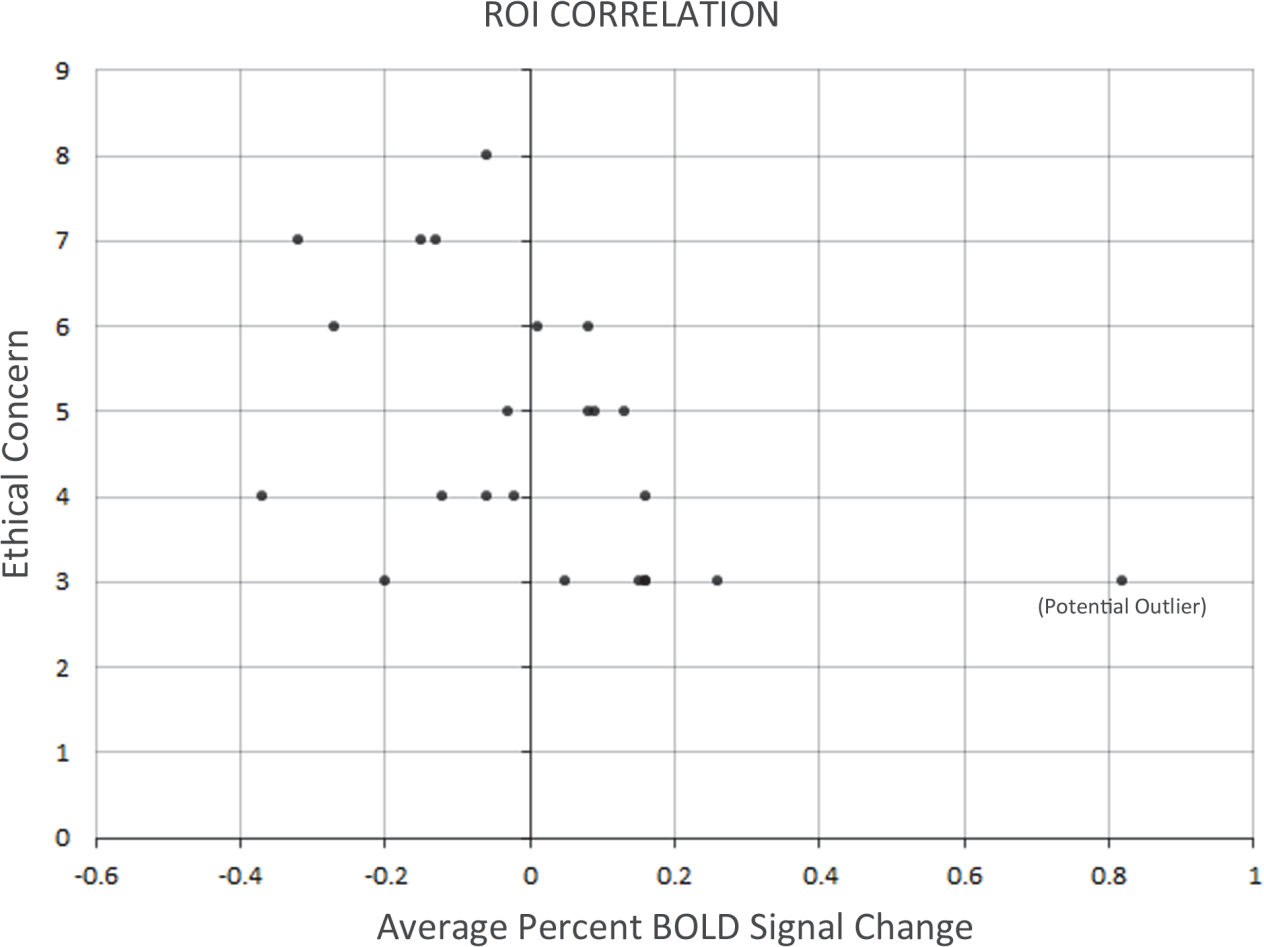
Scatterplot depicting the negative correlation between participants’ scores on the Ethical Concern subscale of the FCQ and their differential neurofunctional activity in the production method > price contrast, *r*(21) = −0.46, *p* < 0.05. When the potential outlier was excluded, the negative correlation survived, *r*(21) = −0.43, *p* < 0.05.

The ROI correlation was performed using only data from participants in Group B. In the production method > price contrast, participants’ scores on the Ethical Concern subscale of the FCQ again correlated negatively and significantly with their differential neurofunctional activity, this time in the 8-mm^3^ volume situated in the left dlPFC, *r*(21) = −0.46, *p* < 0.05 (see Table 1, Fig. 4).

## Discussion

For consumers today, the perceived ethicality of a food’s production method can be as important a purchasing consideration as its price [41–42]. Still, few studies have examined how, neurofunctionally, consumers are making ethical, food-related decisions. In the present study, we examined the neurofunctional correlates of ethical, food-related decision-making, focusing specifically on how consumers’ ethical concern about a food’s production method may relate to how, neurofunctionally, they make decisions whether to purchase that food. To this end, we examined how participants’ scores on the Ethical Concern subscale of the FCQ related to their differential neurofunctional activity when making food-related decisions, specifically, whether to purchase eggs differing in either their price (i.e., high or low) or production method (i.e., with or without the use of cages), but not both. The purpose of this examination was to shed light on the cortical regions facilitating a now common, yet still poorly understood consumer behavior.

Similar cortical regions, including the dlPFC and vmPFC, are thought to facilitate valuation in the contexts of both ethical [65–66] and food-related [67–68] decision-making. We therefore hypothesized, when making decisions whether to purchase eggs based on their production method, as opposed to their price, those participants who took ethical concern into higher consideration would also demonstrate higher differential neurofunctional activity in the dlPFC and vmPFC. To test this hypothesis, we employed a conservative analytical approach. First, we randomly assigned participants to two groups of 23 participants each (viz., Groups A and B). Then, using behavioral and neurofunctional data from participants in Group A, we performed an exploratory, whole-brain correlation for the purpose of identifying potential ROIs. When only one ROI was identified, specifically in the left dlPFC, that ROI then served as the location for a subsequent, confirmatory, ROI correlation using behavioral and neurofunctional data from participants in Group B.

The results of these analyses were, in part, contrary to our hypothesis. When making decisions whether to purchase eggs based on their production method, as opposed to their price, those participants who took ethical concern into higher consideration actually demonstrated *lower* differential neurofunctional activity in the left dlPFC (see Results, Table 1). This suggests, when making ethical, food-related decisions, the more consumers take ethical concern into consideration, the less they may rely on neurofunctional activity in the left dlPFC, possibly because making these decisions is more routine for them, and therefore a more perfunctory process requiring fewer cognitive resources (see [76]). That is, for these consumers, making decisions whether to purchase controversial foods may require less reliance on neurofunctional activity in the cortical regions thought to facilitate such decision-making, as these consumers may make such decisions more frequently, and therefore more fluently (cf. [77–78]).

Although the direction of the correlations we observed was opposite from what we hypothesized, their location in the left dlPFC was consistent with our hypothesis. Interestingly, however, the results of past studies examining the neurofunctional correlates of ethical decision-making have more frequently revealed differential neurofunctional activity in the right dlPFC, as well as the vmPFC [79]. Still, consistent with our results, the results of these studies have also occasionally revealed differential neurofunctional activity in the dlPFC bilaterally (e.g., [53, 65]) and, albeit less frequently, the left dlPFC (e.g., [80–82]), although the results of the study by Harrison et al. [81] revealed differential neurofunctional activity in the left dlPFC more anterior than that revealed by our results. Moreover, when these studies have employed complex, emotional stimuli, their results have only rarely revealed differential neurofunctional activity in the vmPFC [52], which could explain why our results did not reveal differential neurofunctional activity in that cortical region as well.

Perhaps most interestingly, our results are also consistent with those of the aforementioned study by Greene et al. [65] examining, in part, the neurofunctional correlates of “personal and impersonal [ethical] judgment.” In that study, personal ethical judgments were understood to be those required in response to personal ethical dilemmas, that is, ethical dilemmas involving (a) a person acting to harm another directly, (b) the directly harmful action itself, and (c) the person directly harmed by that action. Conversely, impersonal ethical judgments were understood to be those required in response to impersonal ethical dilemmas, that is, ethical dilemmas not involving one or more of the components of personal ethical dilemmas. Greene et al. [65] found participants demonstrated higher differential neurofunctional activity in the dlPFC bilaterally, but particularly in the right dlPFC, when making impersonal ethical judgments, as opposed to personal ones.

Our results offer further support for this finding. In the present study, when making decisions whether to purchase eggs based on their production method, as opposed to their price, participants were not responding to a type of personal ethical dilemma. Making the decision whether to purchase eggs produced with or without the use of cages is unlikely to harm another person directly. Rather, participants were responding to a type of impersonal ethical dilemma, one requiring impersonal ethical judgment. Consistent with the results of the study by Greene et al. [65], our results revealed the left dlPFC to facilitate such ethical judgment, at least in part. Again, they did not reveal the same of the right dlPFC.

The present study did suffer from a few notable limitations. First, any meaningful interpretation of our results depends, in part, on the assumption that participants found the prospect of receiving eggs they had made the decision to purchase sufficiently rewarding to incentivize their non-hypothetical decision-making. Second, it also depends on the assumption that making decisions whether to purchase a food based on its price, as opposed to its production method, does not have an ethical dimension. For example, consumers may perceive foods with high prices as more “unfair” or “unjust” than those with low prices. If this is indeed the case, future studies examining the neurofunctional correlates of ethical, food-related decision-making may benefit from using an experimental paradigm that better isolates the effect on neurofunctional activity of prompting participants to take ethical concern into consideration as they make food-related decisions.

A third limitation to our study lies in the Ethical Concern subscale of the FCQ. Of the FCQ’s 36 items, only three describe food-related attributes associated with its Ethical Concern subscale. Of these three items, only one relates to ethical concern about the environment, and none relates to ethical concern about animal welfare. Although the FCQ remains a valuable measure of respondents’ ethical concern when making food-related decisions, a revised version has been developed to measure more precisely ethical concern about animal welfare and the environment [83]. Unfortunately, this revised version of the FCQ was unknown to us until we had completed collecting behavioral data for the present study. Future studies examining the neurofunctional correlates of ethical, food-related decision-making may benefit from using this version of the FCQ instead.

Intensive animal agriculture, and particularly the use of CAFOs, has wrought serious consequences for animal welfare and the environment [13]. So serious are these consequences that consumers are now willing to pay premiums for foods produced without the use of cages, crates, and other forms of confinement common in CAFOs [42]. However, *how* consumers are making ethical, food-related decisions remains only partially understood. The purpose of the present study was to supplement this understanding using the tools of decision neuroscience, examining the neurofunctional correlates of ethical, food-related decision-making. Our results contribute a modest first step toward a more complete understanding of how the dlPFC may facilitate valuation in this nuanced, but now common decision-making context.

## References

[1] Pollan M. The omnivore’s dilemma: A natural history of four meals New York: Penguin Press; 2002.

[2] Pollan M. In defense of food: An eater’s manifesto New York: Penguin Press; 2008.

[3] Pew Commission on Industrial Farm Animal Production. Putting meat on the table: Industrial farm animal production in America. 2008. Available: http://www.ncifap.org/_images/PCIFAPFin.pdf.

[4] Hribar C. Understanding concentrated animal feeding operations and their impact on communities Bowling Green (OH): National Association of Local Boards of Health; 2010.

[5] MacDonald JM, McBride WD. The transformation of U.S. livestock agriculture (Bulletin No. 43). Washington (DC): United States Department of Agriculture; 2009.

[6] Delgado CL. Rising consumption of meat and milk in developing countries has created a new food revolution. The Journal of Nutrition. 2003;133(11):3907S–3910S. 14672289 10.1093/jn/133.11.3907S

[7] Trewavas A. Malthus foiled again and again. Nature. 2002;418(6898):668–670. 10.1038/nature01013 12167872

[8] Key N, McBride W. The changing economics of U.S. hog production (Report No. 52). Washington (DC): United States Department of Agriculture; 2007.

[9] MacDonald JM, O’Donoghue EJ, McBride WD, Nehring RF, Sandretto CL, Mosheim R. Profits, costs, and the changing structure of dairy farming (Report No. 47). Washington (DC): United States Department of Agriculture; 2007.

[10] MacDonald JM. The economic organization of U.S. broiler production (Bulletin No. 38). Washington (DC): United States Department of Agriculture; 2008.

[11] Marsh JM. U.S. feeder cattle prices: Effects of finance and risk, cow-calf and feedlot technologies, and Mexican feeder imports. Journal of Agricultural and Resource Economics. 2001;26(2):463–477.

[12] Ollinger M, Nguyen SV, Blayney D, Chambers B, Nelson K. Structural change in the meat, poultry, dairy, and grain processing industries (Report No. 3). Washington (DC): United States Department of Agriculture; 2005.

[13] Gurian-Sherman D. CAFOs uncovered: The untold costs of confined animal feeding operations Cambridge (MA): Union of Concerned Scientists; 2008.

[14] Holt DM. Unlikely allies against factory farms: Animal rights advocates and environmentalists. Agriculture and Human Values. 2008;25(2):169–171. 10.1007/s10460-008-9122-4

[15] Ilea RC. Intensive livestock farming: Global trends, increased environmental concerns, and ethical solutions. Journal of Agricultural and Environmental Ethics. 2009;22(2):153–167. 10.1007/s10806-008-9136-3

[16] Thorne PS. Environmental health impacts of concentrated animal feeding operations: Anticipating hazards—Searching for solutions. Environmental Health Perspectives. 2007;115(2):296–297. 10.1289/ehp.8831 17384781 PMC1817701

[17] Tilman D, Cassman KG, Matson PA, Naylor R, Polasky S. Agricultural sustainability and intensive production practices. Nature. 2002;418(6898):671–677. 10.1038/nature01014 12167873

[18] Heederik D, Sigsgaard T, Thorne PS, Kline JN, Avery R, Bønløkke JH, et al Health effects of airborne exposures from concentrated animal feeding operations. Environmental Health Perspectives. 2007;115(2):298–302. 10.1289/ehp.8835 17384782 PMC1817709

[19] Mirabelli MC, Wing S, Marshall SW, Wilcosky TC. Race, poverty, and potential exposure of middle-school students to air emissions from confined swine feeding operations. Environmental Health Perspectives. 2006;114(4):591–596. 10.1289/ehp.8586 16581551 PMC1440786

[20] Burkholder J, Libra B, Weyer P, Heathcote S, Kolpin D, Thorne PS, et al Impacts of waste from concentrated animal feeding operations on water quality. Environmental Health Perspectives. 2007;115(2):308–312. 10.1289/ehp.8839 17384784 PMC1817674

[21] Patel P, Centner TJ. Air pollution by concentrated animal feeding operations. Desalination and Water Treatment. 2010;19(1–3):12–16. 10.5004/dwt.2010.1890

[22] West BM, Liggit P, Clemans DL, Francoeur SN. Antibiotic resistance, gene transfer, and water quality patterns observed in waterways near CAFO farms and wastewater treatment facilities. Water, Air, & Soil Pollution. 2011;217(1):473–489. 10.1007/s11270-010-0602-y

[23] Fiala N. Meeting the demand: An estimation of potential future greenhouse gas emissions from meat production. Ecological Economics. 2008;67(3):412–419. 10.1016/j.ecolecon.2007.12.021

[24] Koneswaran G, Nierenberg D. Global farm animal production and global warming: Impacting and mitigating climate change. Environmental Health Perspectives. 2008;116(5):578–582. 10.1289/ehp.11034 18470284 PMC2367646

[25] Verheul J. Methane as a greenhouse gas: Why the EPA should regulate emissions from animal feeding operations and concentrated animal feeding operations under the Clean Air Act. Natural Resources Journal. 2011;51(1):163–187.

[26] Alvarado CS, Gibbs SG, Gandara A, Flores C, Hurd WW, Green CF. The potential for community exposures to pathogens from an urban dairy. Journal of Environmental Health. 2012;74(7):22–28. 22428319

[27] Green CF, Gibbs SG, Tarwater PM, Mota LC, Scarpino PV. Bacterial plume emanating from the air surrounding swine confinement operations. Journal of Occupational and Environmental Hygiene. 2006;3(1):9–15. 10.1080/15459620500430615 16482973

[28] Greger M, Koneswaran G. The public health impacts of concentrated animal feeding operations on local communities. Family Community Health. 2010;33(1):373–382. 10.1097/fch.0b013e3181c4e22a 20010001

[29] Walker P, Rhubart-Berg P, McKenzie S, Kelling K, Lawrence RS. Public health implications of meat production and consumption. Public Health Nutrition. 2005;8(4):348–356. 10.1079/phn2005727 15975179

[30] Saja K. The moral footprint of animal products. Agriculture and Human Values. 2013;30(2):193–202. 10.1007/s10460-012-9402-x

[31] Thompson PB. Animal ethics and public expectations: The North American outlook. Journal of Veterinary Medical Education. 2010;37(1):13–21. 10.3138/jvme.37.1.13 20378872

[32] McLeod-Kilmurray H. Commoditizing nonhuman animals and their consumers: Industrial livestock production, animal welfare, and ecological justice. Bulletin of Science, Technology & Society. 2012;32(1):71–85. 10.1177/0270467612444585

[33] Boyle LA, Leonard FC, Lynch PB, Brophy P. Effect of gestation housing on behaviour and skin lesions of sows in farrowing crates. Applied Animal Behaviour Science. 2002;76(2):119–134. 10.1016/s0168-1591(01)00211-8

[34] D’Silva J. Adverse impact of industrial animal agriculture on the health and welfare of farmed animals. Integrative Zoology. 2006;1(1):53–58. 10.1111/j.1749-4877.2006.00013.x 21395992

[35] Jensen TB, Toft N, Bonde MK, Kongsted AG, Kristensen AR, Sørensen JT. Herd and sow-related risk factors for mortality in sows in group-housed systems. Preventive Veterinary Medicine. 2012;103(1):31–37. 10.1016/j.prevetmed.2011.09.009 21996451

[36] Kolbe EA. "Won't you be my neighbor?" Living with concentrated animal feeding operations. Iowa Law Review. 2013;99(1):415–443. 10.2139/ssrn.2387639

[37] Norwood FB, Lusk JL. Compassion, by the pound: The economics of farm animal welfare New York: Oxford University Press; 2011.

[38] Frewer LJ, Kole A, Van de Kroon SMA, de Lauwere C. Consumer attitudes towards the development of animal-friendly husbandry systems. Journal of Agricultural and Environmental Ethics. 2005;18(4):345–367. 10.1007/s10806-005-1489-2

[39] Prickett RW, Norwood FB, Lusk JL. Consumer preferences for farm animal welfare: Results from a telephone survey of US households. Animal Welfare. 2010;19(3):335–347.

[40] Harper GC, Makatouni A. Consumer perception of organic food production and farm animal welfare. British Food Journal. 2002;104(3–5):287–299. 10.1108/00070700210425723

[41] Lusk JL, Nilsson T, Foster K. Public preferences and private choices: Effect of altruism and free riding on demand for environmentally certified pork. Environmental & Resource Economics. 2007;36(4):499–521. 10.1007/s10640-006-9039-6

[42] Norwood FB, Lusk JL. A calibrated auction-conjoint valuation method: Valuing pork and eggs produced under differing animal welfare conditions. Journal of Environmental Economics and Management. 2011;62(1):80–94. 10.1016/j.jeem.2011.04.001

[43] Hurley SP, Miller DJ, Kliebenstein JB. Estimating willingness to pay using a polychotomous choice function: An application to pork products with environmental attributes. Journal of Agricultural and Resource Economics. 2006;31(2):301–317.

[44] Tonsor GT, Olynk N, Wolf C. Consumer preferences for animal welfare attributes: The case of gestation crates. Journal of Agricultural and Applied Economics. 2009;41(3):713–730.

[45] Tonsor GT, Wolf C, Olynk N. Consumer voting and demand behavior regarding swine gestation crates. Food Policy. 2009;34(6):492–498. 10.1016/j.foodpol.2009.06.008

[46] Sanfey AG, Loewenstein G, McClure SM, Cohen JD. Neuroeconomics: Cross-currents in research on decision-making. Trends in Cognitive Sciences. 2006;10(3):108–116. 10.1016/j.tics.2006.01.009 16469524

[47] Bogacz R. Optimal decision-making theories: Linking neurobiology with behaviour. Trends in Cognitive Sciences. 2007;11(3):118–125. 10.1016/j.tics.2006.12.006 17276130

[48] Gold JI, Shadlen MN. The neural basis of decision making. Annual Review of Neuroscience. 2007;30(1):535–574. 10.1146/annurev.neuro.29.051605.113038 17600525

[49] Platt ML. Neural correlates of decisions. Current Opinion in Neurobiology. 2002;12(2):141–148. 10.1016/s0959-4388(02)00302-1 12015229

[50] Schall JD. Neural correlates of decision processes: Neural and mental chronometry. Current Opinion in Neurobiology. 2003;13(2):182–186. 10.1016/s0959-4388(03)00039-4 12744971

[51] Smith PL, Ratcliff R. Psychology and neurobiology of simple decisions. Trends in Neurosciences. 2004;27(3):161–168. 10.1016/j.tins.2004.01.006 15036882

[52] Greene J, Haidt J. How (and where) does moral judgment work? Trends in Cognitive Sciences. 2002;6(12):517–523. 10.1016/s1364-6613(02)02011-9 12475712

[53] Heekeren HR, Wartenburger I, Schmidt H, Schwintowski H-P, Villringer A. An fMRI study of simple ethical decision-making. NeuroReport. 2003;14(9):1215–1219. 12824762 10.1097/00001756-200307010-00005

[54] Moll J, de Oliveira-Souza R. Moral judgments, emotions and the utilitarian brain. Trends in Cognitive Sciences. 2007;11(8):319–321. 10.1016/j.tics.2007.06.001 17602852

[55] Pascual L, Rodrigues P, Gallardo-Pujol D. How does morality work in the brain? A functional and structural perspective of moral behavior. Frontiers in Integrative Neuroscience. 2013;7(65):1–8. 10.3389/fnint.2013.00065 24062650 PMC3770908

[56] Bruce AS, Lusk JL, Crespi JM, Cherry JBC, Bruce JM, McFadden BR, et al Consumers’ neural and behavioral responses to food technologies and price. Journal of Neuroscience, Psychology, and Economics. 2014;7(3):164–173. 10.1037/npe0000023

[57] Linder NS, Uhl G, Fliessbach K, Trautner P, Elger CE, Weber B. Organic labeling influences food valuation and choice. NeuroImage. 2010;53(1): 215–220. 10.1016/j.neuroimage.2010.05.077 20570738

[58] Yoon C, Gonzalez R, Bechara A, Berns GS, Dagher AA, Dubé L, et al Decision neuroscience and consumer decision making. Marketing Letters. 2012;23(2):473–485. 10.1007/s11002-012-9188-z

[59] Paulus MP, Hozack N, Zauscher B, McDowell JE, Frank L, Brown GG, et al Prefrontal, parietal, and temporal cortex networks underlie decision-making in the presence of uncertainty. NeuroImage. 2001;13(1):91–100. 10.1006/nimg.2000.0667 11133312

[60] Dorris MC, Glimcher PW. Activity in posterior parietal cortex is correlated with the relative subjective desirability of action. Neuron. 2004;44(2):365–378. 10.1016/j.neuron.2004.09.009 15473973

[61] Huk AC, Meister MLR. Neural correlates and neural computations in posterior parietal cortex during perceptual decision-making. Frontiers in Integrative Neuroscience. 2012;6(86):1–13. 10.3389/fnint.2012.00086 23087623 PMC3467999

[62] Hutcherson CA, Plassmann H, Gross JJ, Rangel A. Cognitive regulation during decision making shifts behavioral control between ventromedial and dorsolateral prefrontal value systems. The Journal of Neuroscience. 2012;32(39):13543–13554. 10.1523/jneurosci.6387-11.2012 23015444 PMC3689006

[63] Kahnt T, Heinzle J, Park SQ, Haynes J-D. Decoding different roles for vmPFC and dlPFC in multi-attribute decision making. NeuroImage. 2011;56(2):709–715. 10.1016/j.neuroimage.2010.05.058 20510371

[64] Sokol-Hessner P, Hutcherson C, Hare T, Rangel A. Decision value computation in DLPFC and VMPFC adjusts to the available decision time. European Journal of Neuroscience. 2012;35(7):1065–1074. 10.1111/j.1460-9568.2012.08076.x 22487036 PMC3325500

[65] Greene JD, Nystrom LE, Engell AD, Darley JM, Cohen JD. The neural bases of cognitive conflict and control in moral judgment. Neuron. 2004;44(2):389–400. 10.1016/j.neuron.2004.09.027 15473975

[66] Koenigs M, Young L, Adolphs R, Tranel D, Cushman F, Hauser M, et al Damage to the prefrontal cortex increases utilitarian moral judgements. Nature. 2007;446(7138):908–911. 10.1038/nature05631 17377536 PMC2244801

[67] Plassmann H, O’Doherty JP, Rangel A. Appetitive and aversive goal values are encoded in the medial orbitofrontal cortex at the time of decision making. The Journal of Neuroscience. 2010;30(32):10799–10808. 10.1523/jneurosci.0788-10.2010 20702709 PMC6634706

[68] Hare TA, Camerer CF, Rangel A. Self-control in decision-making involves modulation of the vmPFC valuation system. Science. 2009;324(5927):646–648. 10.1126/science.1168450 19407204

[69] Hare TA, Malmaud J, Rangel A. Focusing attention on the health aspects of foods changes value signals in vmPFC and improves dietary choice. The Journal of Neuroscience. 2011;31(30):11077–11087. 10.1523/jneurosci.6383-10.2011 21795556 PMC6623079

[70] Steptoe A, Pollard TM, Wardle J. Development of a measure of the motives underlying the selection of food: The food choice questionnaire. Appetite. 1995; 25(3):267–284. 8746966 10.1006/appe.1995.0061

[71] Bruce AS, Lepping RJ, Bruce JM, Cherry JBC, Martin LE, Davis AM, et al Brain responses to food logos in obese and healthy-weight children. The Journal of Pediatrics. 2013;162(4):759–764. 10.1016/j.jpeds.2012.10.003 23211928

[72] Amaro E, Barker GJ. Study design in fMRI: Basic principles. Brain and Cognition. 2006;60: 220–232. 10.1016/j.bandc.2005.11.009 16427175

[73] Talairach J, Tournoux P. Co-planar stereotaxic atlas of the human brain New York: Thieme; 1988.

[74] Friston KJ, Holmes AP, Poline J-B, Grasby PJ, Williams SCR, Frackowiak RSJ, et al Analysis of fMRI time-series revisited. NeuroImage. 1995;2(1):45–53. 10.1006/nimg.1995.1007 9343589

[75] Vul E, Harris C, Winkielman P, Pashler H. Puzzlingly high correlations in fMRI studies of emotion, personality, and social cognition. Perspectives on Psychological Science. 2009;4(3):274–290. 10.1111/j.1745-6924.2009.01125.x 26158964

[76] Rypma B, Berger JS, Prabhakaran V, Bly BM, Kimberg DY, Biswal BB, et al Neural correlates of cognitive efficiency. NeuroImage. 2006;33(3):969–979. 10.1016/j.neuroimage.2006.05.065 17010646

[77] Gray JR, Chabris CF, Braver TS. Neural mechanisms of general fluid intelligence. Nature Neuroscience. 2003;6(3):316–322. 10.1038/nn1014 12592404

[78] Newman SD, Carpenter PA, Varma S, Just MA. Frontal and parietal participation in problem solving in the Tower of London: fMRI and computational modeling of planning and high-level perception. Neuropsychologia. 2003;41(12):1668–1682. 10.1016/s0028-3932(03)00091-5 12887991

[79] Moll J, Zahn R, de Oliveira-Souza R, Krueger F, Grafman J. The neural basis of human moral cognition. Nature Reviews Neuroscience. 2005;6(10):799–809. 10.1038/nrn1768 16276356

[80] Cikara M, Farnsworth RA, Harris LT, Fiske ST. On the wrong side of the trolley track: Neural correlates of relative social valuation. Social Cognitive and Affective Neuroscience. 2010;5(4):404–413. 10.1093/scan/nsq011 20150342 PMC2999760

[81] Harrison BJ, Pujol J, Soriano-Mas C, Hernández-Ribas R, López-Solà M, Ortiz H, et al Neural correlates of moral sensitivity in obsessive-compulsive disorder. Archives of General Psychiatry. 2012;69(7):741–749. 10.1001/archgenpsychiatry.2011.2165 22752238

[82] Schleim S, Spranger TM, Erk S, Walter H. From moral to legal judgment: The influence of normative context in lawyers and other academics. Social Cognitive and Affective Neuroscience. 2011;6(1):48–57. 10.1093/scan/nsq010 20194515 PMC3023080

[83] Lindeman M, Väänänen M. Measurement of ethical food choice motives. Appetite. 2000;34(1):55–59. 10.1006/appe.1999.0293 10744892

